# S-acylation of Ca^2+^ transport proteins: molecular basis and functional consequences

**DOI:** 10.1042/BST20230818

**Published:** 2024-02-13

**Authors:** Raphaël Néré, Sana Kouba, Amado Carreras-Sureda, Nicolas Demaurex

**Affiliations:** Department of Cell Physiology and Metabolism, University of Geneva, Geneva, Switzerland

**Keywords:** Ca^2+^ signalling, Ca^2+^-handling proteins, S-acylation, S-palmitoylation

## Abstract

Calcium (Ca^2+^) regulates a multitude of cellular processes during fertilization and throughout adult life by acting as an intracellular messenger to control effector functions in excitable and non-excitable cells. Changes in intracellular Ca^2+^ levels are driven by the co-ordinated action of Ca^2+^ channels, pumps, and exchangers, and the resulting signals are shaped and decoded by Ca^2+^-binding proteins to drive rapid and long-term cellular processes ranging from neurotransmission and cardiac contraction to gene transcription and cell death. S-acylation, a lipid post-translational modification, is emerging as a critical regulator of several important Ca^2+^-handling proteins. S-acylation is a reversible and dynamic process involving the attachment of long-chain fatty acids (most commonly palmitate) to cysteine residues of target proteins by a family of 23 proteins acyltransferases (zDHHC, or PATs). S-acylation modifies the conformation of proteins and their interactions with membrane lipids, thereby impacting intra- and intermolecular interactions, protein stability, and subcellular localization. Disruptions of S-acylation can alter Ca^2+^ signalling and have been implicated in the development of pathologies such as heart disease, neurodegenerative disorders, and cancer. Here, we review the recent literature on the S-acylation of Ca^2+^ transport proteins of organelles and of the plasma membrane and highlight the molecular basis and functional consequence of their S-acylation as well as the therapeutic potential of targeting this regulation for diseases caused by alterations in cellular Ca^2+^ fluxes.

## Introduction

### Ca^2+^ signalling in physiology

Ca^2+^ ions act as secondary messengers in eukaryotic organisms to co-ordinate a wide range of physiological functions, including neurotransmission, muscle contraction, immune regulation, and fertilization [1–5]. The concentration of Ca^2+^ ions at rest is maintained ∼ 100 nM in the cytoplasm, five orders of magnitude lower than the extracellular Ca^2+^ concentration of 1–2 mM. Inside cellular compartments, Ca^2+^ ranges between 100 and 200 nM in mitochondria, whereas in the endoplasmic/sarcoplasmic reticulum (ER/SR), Ca^2+^ levels are maintained at higher concentration ranging from 100 to 500 µM. Ca^2+^ levels are controlled in both time and space to prevent cytotoxicity and enable appropriate responses to various stimuli. This task is achieved through a repertoire of Ca^2+^ channels, pumps and exchangers, all of which are under the control of various Ca^2+^ sensing and auxiliary proteins. These Ca^2+^ handling proteins are susceptible to a range of post-translational modifications (PTMs) that significantly influence their operational characteristics, activity levels, degradation pathways, subcellular localization, and interactions with other cellular components. These intricate modifications encompass oxidative and reductive modifications, glycosylation, ubiquitination, and acylation [6–8]. Here, we summarize recent studies reporting the S-acylation of Ca^2+^ transporters, detailing the biochemical assays used, the residues and enzymes identified, and the functional consequence of this PTM for calcium fluxes and cellular functions.

### S-acylation: definition, characteristics and mechanism

**Figure 1.**
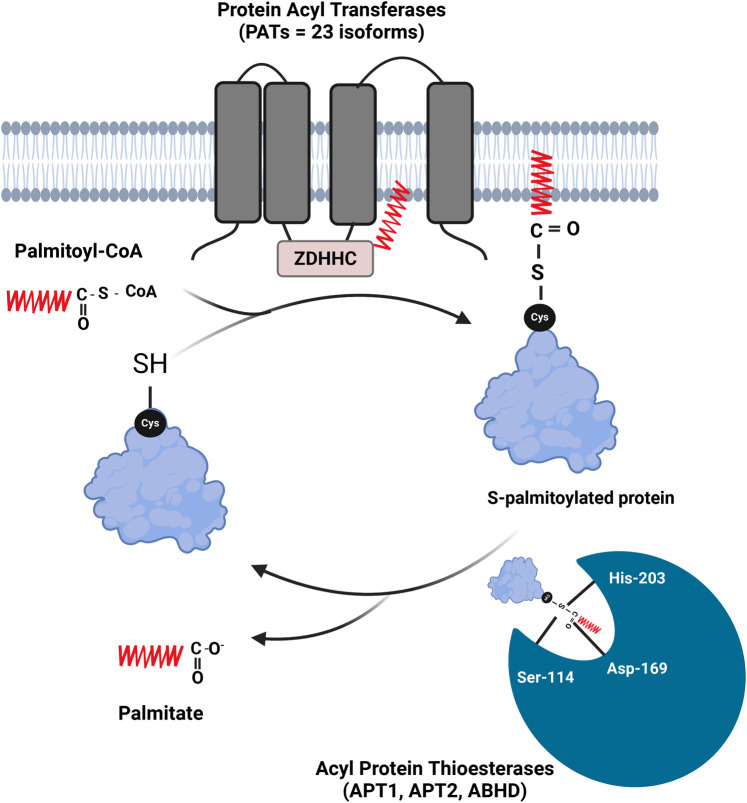
Protein S-acylation cycle. The palmitoyl group is transferred from the palmitoyl-Coenzyme A to the free thiol groups of cysteines by zDHHC–PAT enzymes (typically with 4, or 6, transmembrane domains, with the catalytic DHHC domain located in a cytosolic loop). The thioester-linked fatty acid is then removed by protein thioesterases (APTs) to regenerate free thiol groups (Created with Biorender.com).

S-acylation (also known as S-palmitoylation) involves the formation of a thioester bond between a fatty acid molecule and a cysteine residue in a protein, linking the sulfur atom of the cysteine to the carbonyl group of the fatty acid (Figure 1). This bond is reversible and allows the dynamic attachment and detachment of the fatty acid to the protein, influencing the protein's function and cellular localization. We refer to this process as S-acylation throughout this review. S-acylation (Figure 1) is catalysed by enzymes with multiple transmembrane domains (four or six) bearing a conserved cysteine-rich region between transmembrane domains 2 and 3. This aspartate-histidine-histidine-cysteine (zDHHC) motif [9] defines a group of zDHHC–palmitoyl acyltransferases (zDHHC–PATs) of which 23 isoforms exist in humans [10]. The zDHHC domain contains a catalytic triad with the initial Asp polarizing the second His, which acts as a base to extract a proton from the terminal Cys to turn this cysteine into a thiolate nucleophile. This nucleophile group can attack the α-carbon of the fatty acyl-coenzyme A (CoA) thioester carbonyl molecule, resulting in acylated zDHHC–PAT. The S-acylated zDHHC–PAT can then bind its protein substrate and transfer the acyl group [11]. Protein de-acylation (de-palmitoylation) involves the hydrolysis of the thioester bond, a process greatly accelerated by the two main acyl-protein thioesterases APT1 and APT2. APT enzymes contain a serine-histidine-aspartic acid catalytic triad that catalyses the breaking of thioester bonds, a long hydrophobic tunnel accommodating the attached lipid [12], and a lid-loop covering the catalytic site and determining the product release rate of the enzyme [13]. Another family of proteins called ABHD17 can contribute to the de-palmitoylation of Ras-family proteins [14],and is a target for anti-cancer drugs [15]. The following section summarizes studies reporting the S-acylation of Ca^2+^ channels, exchangers, and pumps that control the fluxes of Ca^2+^ ions at the plasma membrane and in intracellular organelles.

## S-acylation of Ca^2+^ channels, pumps and exchangers

### Voltage-gated Ca^2+^ channels

#### CaV1.2

**Figure 2.**
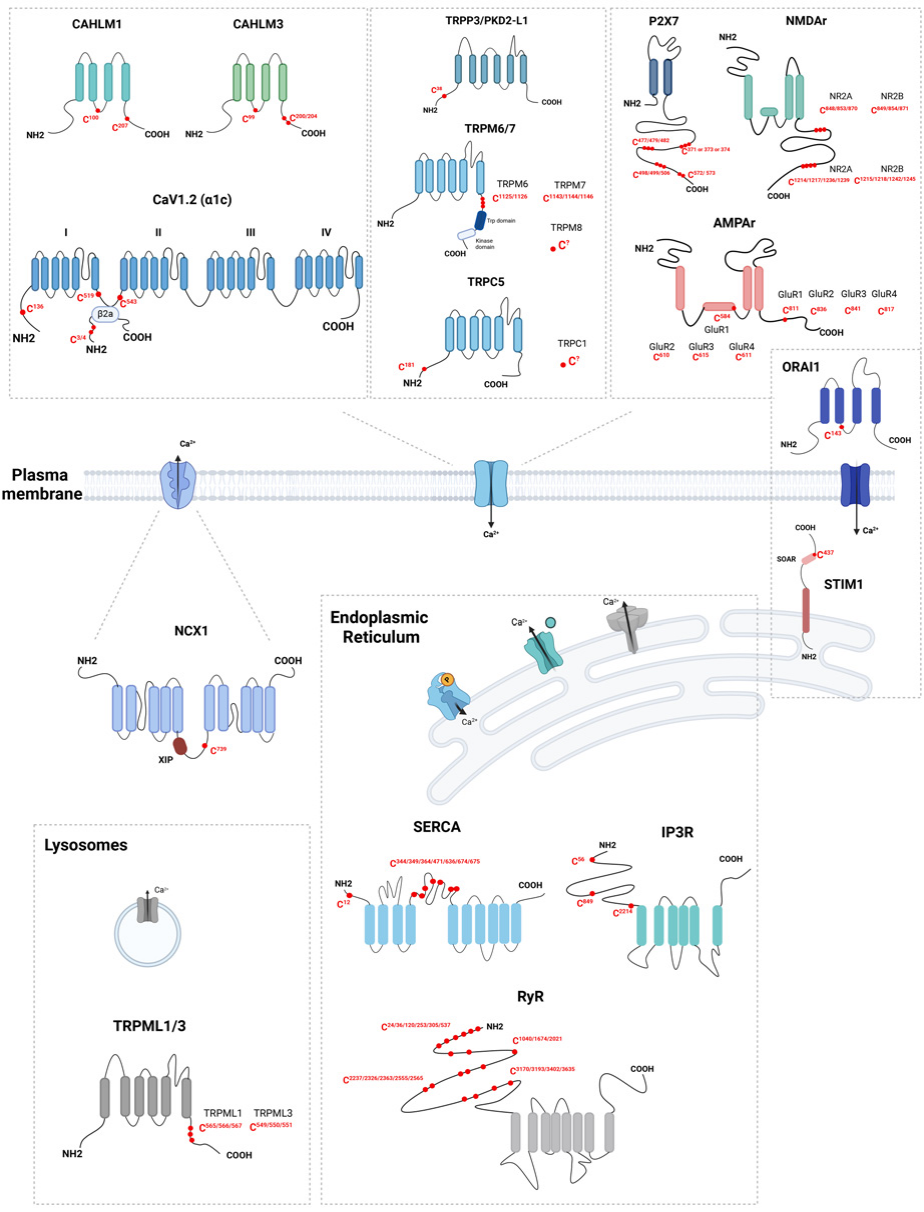
S-acylation of Ca2+ handling proteins. (Created with Biorender.com).

Voltage-gated Ca^2+^ channels (Ca_v_) are composed of multiple subunits including a pore-forming α subunit and an accessory β subunit (Table 1). They control excitation-contraction coupling in muscle, neurotransmitter release in neurons, and insulin secretion by pancreatic beta cells [16]. The pore-forming α1C subunit of Ca_v_1.2, a L-type voltage-dependent Ca^2+^ channel encoded by the *CACNA1C* gene, was recently reported to be S-acylated in ventricular myocytes using resin-assisted capture of acylated proteins (Acyl-RAC) [17]. Acyl-RAC, along with acyl-biotin exchange and acyl-PEG exchange, rely on the breaking of disulfuric bonds, blocking of the free cysteines and cleaving of the S-acylated cysteine [18]. The freed cysteine can then be captured, and the protein analysed using Western-Blot or mass spectrometry. The S-acylation sites were mapped to the channel N terminus (C136) and to the linker between the two first domains of the protein (C519 and C543) (Figure 2). When expressed in HEK-293 cells, α1C bearing the triple substitution C136/519/543A was not acylated and mediated whole-cell currents with reduced voltage-sensitivity (+10-mV positive shift) but preserved activation kinetics. The triple cysteine substitution reduced Ca^2+^ transients recorded in the presence of nifedipine when introduced in a dihydropyridine-resistant α1C in cardiomyocytes. Palmitoylation therefore controls the voltage-sensitivity of Ca_v_1.2 and this regulatory mechanism could be targeted pharmacologically to prevent fatal cardiac arrythmia [17,19].

**Table 1.** S-acylation of Ca^2+^ transporters

Gene	Protein	Function	Localization	Modified cysteine(s)	Modifying enzyme(s)	Technique(s)	Functional consequences of S-acylation	References
*CACNA1C*	α1C subunit of CaV1.2	Voltage-gated Ca^2+^ channel (pore-forming subunit)	PM	C136, C519 and C543	**—**	Acyl-PEG5-exchange and Acyl-RAC	Increased Ca^2+^ channel activity	[17]
*CACNB2*	β2a subunit of CaV1.2	Voltage-gated Ca^2+^ channel (regulatory subunit)	Cytosol	C3 and C4	**—**	Radiolabelling 3H-palmitic acid incorporation	Increased Ca^2+^ channel activity by tethering α1C in the PM	[21,22,24]
*CALHM1*	CALHM1	Voltage-gated Ca^2+^ and ATP permeable channel (pore-forming subunit)	PM	C100 and C207	ZDHHC3, ZDHHC7, ZDHCC20	Acyl-biotin exchange and on-bead click chemistry	Decreased activity of homomeric CALHM1 channels	[27]
*CALHM3*	CALHM3	Voltage-gated Ca^2+^ and ATP permeable channel (regulatory subunit)	PM	C99, C200 and C204	ZDHHC3 and ZDHHC15	Acyl-biotin exchange and on-bead click chemistry	Increased activity of heteromeric CALHM1/3 channels	[28]
*TRPC1*	TRPC1	Cation channel	PM	**—**	ZDHHC3 and ZDHHC7	Radiolabelling 3H-palmitic acid incorporation	**—**	[31]
*TRPC5*	TRPC5	Cation channel	PM	C181	**—**	click chemistry with biotin-azide and 17-ODYA	Increased channel activity by enhancing ER to PM traffic	[34]
*TRPM6*	TRPM6	Cation channel	PM	C1125 and C1126	**—**	Acyl-RAC	**—**	[37]
*TRPM7*	TRPM7	Cation channel	PM	C1143, C1144 and C1146	ZDHHC17 in Golgi and ZDHCC5 at PM	Acyl-RAC	Increased channel activity by enhancing Golgi to PM traffic	[37]
*TRPM8*	TRPM8	Cation channel	PM	**—**	ZDHHC3 and ZDHHC7	Radiolabelling 3H-palmitic acid incorporation	**—**	[31]
*MCOLN1*	TRPML1	Ca^2+^ release channel	Endosomes, lysosomes	C565, C566 and C567	**—**	Radioactive palmitate labelling	Dynamic trafficking to the cell surface and increased efficiency of endocytosis.	[40]
*MCOLN3*	TRPML3	Ca^2+^ release channel	Endosomes, lysosomes, autophagosomes	C550, C551 and C549	ZDHHC1 and ZDHHC11	Palmitoylation assay, Metabolic labelling, MS	Dynamic trafficking and cellular function	[44]
*PKD2L1*	TRPP3	Cation channel	PM	C38	**—**	Acyl-Biotin exchange	Increased channel activity by enhancing PM tethering	[48]
*ORAI1*	ORAI1	Store-operated Ca^2+^ channel	PM	C143	ZDHHC3, ZDHHC7, ZDHHC20	Acyl-PEG5-exchange and Radiolabelling 3H-palmitic acid incorporation	Increased Ca^2+^ channel activity by targeting Orai1 to lipid rafts and immune synapses	[57,58]
*STIM1*	STIM1	ER Ca^2+^ sensor, activator of ORAI1	ER	C437	ZDHHC20	Acyl-RAC	Increased colocalization with Orai1 and Ca^2+^ entry	[62]
*GRIN2A and GRIN2B*	NMDA receptor subunits NR2A and NR2B	Glutamate ionotropic receptor	PM	Cluster 1: NR2A (C848, 853, and 870) and NR2B (C849, 854, and 871) Cluster 2: NR2A (C1214, 1217, 1236, and 1239) and NR2B (C1215, 1218, 1242, and 1245)	zDHHC3	Radiolabelling 3H-palmitic acid incorporation	Cluster 1: Increased surface expression of NMDAR due to enhanced phosphorylation of tyrosine-based internalization motifs by Src. Cluster 2: decreased surface expression and accumulation in the Golgi apparatus	[50]
*GRIA1, GRIA2, GRIA3 and GRIA4*	AMPA receptor subunits GluR1, GluR2, GluR3, and GluR4	Glutamate ionotropic receptor	PM	C-term (GluR1-C811; GluR2-C836; GluR3-C841; GluR4-C817 and TMD2 (GluR1C585, GluR2C610, GluR3C615, and GluR4C611)	zDHHC3	Radiolabelling 3H-palmitic acid incorporation	TMD2: Reduced surface expression and accumulation in the Golgi apparatus. C-term reduced interaction with the 4.1N protein and receptor internalization	[51]
*P2RX7*	P2X7	Purinergic ionotropic ligand-gated channel	PM	Group 3 (C371 or 373 or 374); Group 5 (C477, C479 and C482); Group 6 (C498, C499, C506); Group 7 (C572 and C573)	**—**	Radiolabelling 3H-palmitic acid incorporation	Increased channel activity by enhancing ER to PM traffic	[55]
*SLC8A1*	NCX1	Sodium-Calcium exchanger	PM	C739	ZDHHC5 APT-1	Acyl-RAC	Decreased exchanger activity by increasing endogenous inhibitory interactions	[65]
*ATP2A1*	SERCA1	Ca^2+^ pump	ER	C12, C344, C349, C364, C471, C636, C674 and C675	**—**	Acyl-RAC, LC–MS/MS	**—**	[68]
*ITPR1*	IP3R	Receptor and Ca^2+^ release channel	ER	C56 (2214) and C849	ZDHHC6	Mass spectroscopy, acyl-biotin exchange	Increased IP3R protein levels and Ca^2+^ release activity	[72]
*RYR1*	RyR1	Ca^2+^ release channel	ER	C24, C36, C253, C305, C537, C1040, C1674, C2021, C2237, C2326, C2363, C2555, C2565, C3170, C3193, C3402 and C3635	**—**	Acyl-RAC, LC–MS/MS, palmitate turnover assay	Increased RYR1 Ca^2+^ release activity	[68]

The β subunits, encoded in four genes with additional diversity generated by alternative splicing, function as regulators of Ca_v_ channel trafficking and gating. Mutations in Ca_v_β2, encoded by the *CACNB2* gene, are associated with mental disorders and cardiovascular diseases [20]. S-acylation of two N-terminal cysteines (C3 and C4) targets Ca_v_β2 to the plasma membrane (Figure 2) and Ca_v_ channels containing an S-acylation-deficient β2a subunit (C3S/C4S) exhibit reduced currents amplitude and lack the distinguishing biophysical properties of Ca_v_β2a channels [21–23]. CACNB2 is highly expressed in human pancreatic islets and expression of Ca_v_β2a but not of its non-acylated C3S/C4S mutant increased surface expression of the pore-forming subunit Ca_v_1.2α1 in insulin-secreting β-cells, increasing basal Ca^2+^ levels and inducing apoptosis [24].

S-acylation thus regulates the activity of Ca_v_1.2 channels by controlling both the surface expression of accessory subunits and the voltage sensitivity of pore-forming subunits.

#### CALHM1

The Ca^2+^ homeostasis modulator 1 (CALHM1, encoded by the *CALHM1* gene) is a voltage-gated, ATP- and Ca^2+^-permeable channel (Table 1) that mediates sweet, bitter and umami perception in taste bud cells [25] and whose deficiency impairs memory flexibility in mice [26]. CALHM1 was shown to be S-acylated by acyl-biotin exchange and to incorporate palmitate by metabolic labelling and click chemistry, establishing that the channel is S-acylated [27]. Combined substitution of two cysteine residues, C100 and C207 (Figure 2), but not substitution or either residue individually, prevented CALHM1 S-acylation and increased ATP release by the overexpressed channel. These residues are located within the juxta-membrane regions of CALHM1 third and fourth transmembrane domain and appear to function as compensatory sites for S-acylation. The C100S/C207S mutation reduced CALHM1 association with detergent-resistant membranes without impacting channel synthesis, degradation, or cell surface expression. zDHHC3, 7 and 20, but none of the other 20 acyltransferases screened, enhanced CALHM1 S-acylation when overexpressed. These three enzymes co-immunoprecipitated with CALHM1-FLAG and decreased CALHM1 S-acylation when down-regulated, establishing that they mediate CALHM1 S-acylation. Endogenous CALHM1 tagged with the V5 epitope was detected by acyl-biotin exchange in taste cells of mice, highlighting that the channel is S-acylated *in vivo*. When expressed in *Xenopus* oocytes, the non-acylated C100S/C207S channel had an increased conductance and a voltage sensitivity shifted by −20 mV, activating at more negative voltages and with faster activation kinetics. S-acylation thus targets CALHM1 to lipid rafts and alters its voltage dependence to down-regulate the channel activity.

#### CALHM3

Heteromeric CALHM1/3 channels also undergo S-acylation [28]. Unlike CALHM1, CALHM3 by itself cannot form a functional channel but enhances the trafficking and gating of CALHM1. Using the approaches detailed above, CALHM3 was shown to be reversibly S-acylated at three intracellular cysteine residues (C99, C200, and C204) (Figure 2) and zDHHC3/zDHHC15 identified as the acylating enzymes. The acylation-deficient C99/200/204S protein retained the ability to enhance the surface expression of co-expressed CALHM1, but heteromeric CALHM1/3 channels bearing the triple mutation had reduced activity compared with wild-type heteromeric channels. It appears therefore that S-acylation of CALHM3 positively regulates the activity of heteromeric CALHM1/3 channels, while the S-acylation of CALHM1 negatively regulates its activity in monomeric channels. This differential regulation occurs without changes in the surface expression of either protein and likely reflects alterations in channel gating. The contribution of CALHM1 S-acylation to the activity of heteromeric CALHM1/3 channels remains to be established.

### Transient receptor potential channels

#### TRPC1

The TRPC1 channel, the first cloned mammalian transient receptor potential (TRP) channel encoded by the *TRPC1* gene, is a non-selective cation channel that regulates Ca^2+^ influx in response to cell surface receptor activation (Table 1). TRPC1 is widely expressed in humans and is present in diverse cellular compartments, including the ER, plasma membrane, and intracellular vesicles. Genetic and functional studies have linked TRPC1 to diabetic nephropathy, Parkinson's disease, and cancer [29]. *In silico* screening with CSS-Palm 2.0, a freely available software for predicting S-acylation sites relying on clustering and scoring of putative S-acylation patterns [30], identified seventeen novel palmitoyl substrates, of which 10, including the TRPC1 ion channel, were confirmed to be acylated by metabolic incorporation of radioactive palmitate. The S-acylation of TRPC1 and all other tested substrates was attributed to the Golgi-localized zDHHC3 enzyme, as determined using the DHHC palmitoylating enzyme library [31]. However, the effect of this S-acylation has not been yet investigated.

#### TRPC5

TRPC5, encoded by the *TRPC5* gene, is a Ca^2+^-permeable cation channel (Table 1) predominantly expressed in the central nervous system (CNS) with limited expression in the kidney and cardiovascular system. TRPC5 is activated following engagement of surface receptors linked to Gq and phospholipase C, and/or Gi proteins and has been linked genetically to anxiety, seizures, and cold sensing [32]. TRPC5 undergoes PTMs, including S-glutathionylation at C176 and C178, leading to excess Ca^2+^ influx and increased Ca^2+^-dependent apoptosis in the striatum of Huntington's disease (HD) [33]. More recently, C181 in TRPC5 N-terminal residue was identified as critical for its function. Utilizing click chemistry with biotin-azide and 17-octadecynoid acid (17-ODYA), S-acylation at C181 was shown to stabilize the tetrameric assembly of TRPC5 during its trafficking to the plasma membrane. Treatment with the S-acylation inhibitor 2-BP significantly reduced TRPC5 channel activity and surface expression [34]. S-acylation thus enhance the membrane stability of TRPC, providing a therapeutic strategy to mitigate TRPC5 toxicity in HD.

#### TRPM7 and TRPM6

The dual-function TRPM7 channel/kinase, encoded by the *TRPM7* gene, combines a membrane cation channel (Table 1) with an intracellular kinase domain and is essential for embryonic development in mice [35]. TRPM7 is ubiquitously expressed and regulates physiological processes such as cell growth, adhesion, and proliferation and has been associated with cancer proliferation, neuronal cell death, and other pathologies [36]. The ion channel region of TRPM7 catalyses the fluxes of divalent cations (Mg^2+^ and Ca^2+^) across cell membranes, while its kinase region regulates gene expression through histone phosphorylation. TRPM7 resides both at the plasma membrane and within intracellular vesicles, where it controls the uptake and release of zinc. Using resin-assisted capture, TRPM7 was found to be S-acylated in cardiovascular tissues from mice, rats, and humans [37]. The S-acylation site was mapped to a cluster of cysteines on the Trp domain at the C-terminal (C1143, C1144, and C1146) (Figure 2) and the closely related TRPM6 channel was also S-acylated at this site (on C1125 and C1126) (Figure 2). Mutation of the three cysteines of TRPM7 to alanine prevented ER export and non-acylated TRPM7 chimaera or Cys-mutants remained sequestered within intracellular vesicles, had reduced surface expression, and reduced Ca^2+^ uptake. Mutation of TRPM7 residues predicted to bind the Golgi-resident enzyme zDHHC17 reduced TRPM7 S-acylation, while re-expression of the surface-resident zDHHC5 enhanced TRPM7 S-acylation. TRPM7 thus appears to be sequentially S-acylated in the ER, Golgi, and PM, and to depend on S-acylation to reach the plasma membrane and to mediate Ca^2+^ fluxes. The cluster of cysteines is retained in many TRPV and TRPC channels, raising the possibility that this regulation is shared within the TRP superfamily.

#### TRPM8

TRPM8, encoded by the *TRPM8* gene, is a cation channel (Table 1) with permeability to both monovalent and divalent cations. It serves as a sensor for cold and is activated by drops in temperature, organic agents (menthol and icilin), voltage, pressure, and osmolality [38]. *In silico* screening with CSS-Palm 2.0 identified TRPM8 as S-acylation substrate, a prediction subsequently validated through metabolic incorporation of radioactive palmitate, and the Golgi-localized zDHHC3 enzyme as mediating its S-acylation. The impact of TRPM8 S-acylation remains unexplored [31].

#### TRPML1

The mucolipin TRP Cation Channel 1 (TRPML1, encoded by the *MCOLN1* gene) is a non-selective cation channel that mediates Ca^2+^ release from late endosomes and lysosomes (Table 1). Mutations in *MCOLN1* have been linked to mucolipidosis type IV, a lysosomal storage disorder characterized by severe neurological and ophthalmologic abnormalities [39]. Using palmitate labelling and cysteine mutagenesis, TPRML1 was shown to be acylated on three consecutive cysteines at position 565–567 (Figure 2). Full length channels bearing the triple Cys substitution failed to internalize after stimulation, indicating that S-acylation promotes TRPML1 endocytosis [40]. S-acylation of TRPML1 was enhanced following stimulation of gastric parietal cells with histamine, suggesting that dynamic S-acylation of TRPML1 might target TRPML1 to specific lipid microdomains during activation of parietal cells [41].

#### TRPML3

TRPML3, also known as Mucolipin-3, encoded by the *MCOLN3* gene, is an endolysosomal cation channel (Table 1) involved in the regulation of endosomal Ca^2+^ and pH and in autophagosome biogenesis [42]. Gain-of-function mutations in TRPML3 cause deafness and behavioural alterations in mice while Trpml3-deficient mice exhibit lung emphysema due to impaired clearance of elastase by alveolar macrophages [43]. Using metabolic labelling and mass spectroscopy, TRPML3 was shown to undergo S-acylation on three C-terminal cysteines, C550, C551, and C549 (Figure 2), a modification reduced by zDHHC1 or zDHHC11 silencing. Serine substitution of these three cysteines did not impair channel function or subcellular localization but reduced its surface expression and prevented TRPML3 trafficking to autophagic structures, suggesting that S-acylation controls TRPML3 trafficking between subcellular compartments [44].

#### PKD2-L1

The polycystin proteins (PC, PKD), linked to polycystic kidney diseases, comprise a class of 6-transmembrane channel proteins (PKD2, PKD2-L1, PKD2-L2; TRPPs) that form Ca^2+^-permeable channels in primary cilia to drive hedgehog signalling during vertebrate development [45,46]. PKD2-L1 (polycystin-2L1, polycystin-L, TRPP2, formerly TRPP3, encoded by the *PKD2L1* gene) is a non-selective cation channel (Table 1) activated by the washout of extracellular acid (acid-evoked off response), highly expressed in the tongue taste receptor cells. PKD2-L1 implication in sour taste detection was recently ruled out by the identification of loss of function genetic variants in Gumuz Ethiopians who had normal sour recognition despite a complete loss of channel function [47]. Human PKD2-L1 was found by acyl-biotin exchange to be S-acylated at C38 (Figure 2) and inhibition of S-acylation with 2-BP or alanine substitution of C38 reduced PKD2-L1-mediated Ca^2+^ currents in *Xenopus* oocytes [48]. A GFP-tagged N-terminal domain bearing the C38A mutation failed to decorate the plasma membrane when expressed in HEK-293 cells, indicating that S-acylation contributes to membrane anchoring. Whether S-acylation contributes to the targeting of PKD2-L1 to cilia or to its function in gustatory receptors cells is unknown.

### Ligand-gated ionotropic ion channels

#### NMDA receptors

The *N*-methyl-d-aspartate (NMDA) receptor, a type of glutamate receptor, functions as the primary excitatory neurotransmitter in the human brain. It is permeable to Ca^2+^, Na^+^, and K^+^ and its permeability to ions is strongly dependent on the composition of the subunit (Table 1). NMDA receptors underly neuronal processes such as long-term potentiation, synaptic plasticity, and memory formation. Excessive stimulation of NMDA receptors cause excitotoxicity and contributes to the pathophysiology of severe diseases, including epilepsy and Alzheimer's disease [49]. Using metabolic incorporation of radioactive palmitate, NR2A and NR2B subunits of the NMDA receptor were shown to exhibit two distinct clusters of S-acylation sites in their C-terminal region. The first cluster consists of three conserved cysteines located in the juxta-membrane region just beyond transmembrane domain 4; C848, C853, and C870 in NR2A and C849, 854, and 871 in NR2B (Figure 2). S-acylation within this cluster enhances the phosphorylation of tyrosine-based internalization motifs by Src family protein kinases, increasing the surface expression of NMDA receptors [50]. The second cluster comprises four conserved cysteines in the middle of the C-terminus, C1214, C1217, C1236, and C1239 in NR2A and C1215, C1218, C1242, and C1245 in NR2B (Figure 2). S-acylation of the second cluster, facilitated by a distinct palmitoyl transferase known as zDHHC3, a Golgi-specific protein with a DHHC zinc finger domain exhibiting PAT activity towards both AMPA [51] and GABAA receptors [52], causes the receptors to accumulate in the Golgi apparatus and reduces receptor surface expression [50]. These findings indicate that S-acylation of NR2 subunits has a dual role, differentially modulating receptor trafficking and hence synaptic plasticity.

#### AMPA receptors

The α-amino-3-hydroxy-5-methyl-4-isoxazole propionate (AMPA) receptor, a ligand-gated cation channel (Table 1), mediates the rapid phase of excitatory postsynaptic currents in the CNS [53]. Comprising four subunits (GluR1, GluR2, GluR3, and GluR4, encoded by the *GRIA1-4* genes), the AMPA receptor undergoes various PTMs. S-acylation of two distinct sites was shown to regulate AMPA receptor localization [51]. Using radiolabeled palmitic acid, two cysteines were identified by mutagenesis as acylated (Figure 2), one in the second transmembrane domain and another in the C terminal domain of GluR1-4. Mutation of these cysteines to serines did not disrupt the assembly of GluR1/GluR2 heteromers expressed in HEK-293 cells or alter their biophysical properties. Enforced expression of Golgi-resident zDHHC3 enhanced the S-acylation of the TM2 cysteines (Figure 2) and reduced surface expression of AMPA receptors by promoting their accumulation in the Golgi apparatus. In contrast, mutation of the C-terminal cysteine did not impact surface expression of AMPA receptor subunits but enhanced the association of GluR1 with the 4.1N protein, which stabilizes receptors at the cell surface by interacting with the actin cytoskeleton. Stimulation of cortical cultured neurons with glutamate promoted the de-acylation of the AMPA receptor, linking receptor S-acylation to its activity. These data indicate that S-acylation of AMPA receptor subunits modulates receptor trafficking and may be regulated by synaptic activity.

#### P2X7 receptor

The purinergic ionotropic P2X7 receptor (P2X7R, encoded by the *P2RX7* gene) is a cation channel (Table 1) gated by ATP involved in inflammation and neuropathic pain, expressed in haematopoietic, epithelial, and neuronal cells [54]. P2X7R was shown to accumulate tritiated palmitate via thioester bonds and to associate with detergent-resistant membranes in a 2-BP but ATP-independent manner in HEK-293 cells and primary macrophages [55]. Among 16 potential sites, 4 clusters of cysteine residues in the channel C-terminal domain were involved in S-acylation, the substitution of juxta-membrane residues, C371, C373, and C374 (Figure 2), reducing and the substitution of distal residues abrogating palmitic acid incorporation. The S-acylation-defective P2X7R mutants did not associate with detergent-resistant membranes, had reduced half-life and cell surface expression, increased proteasomal degradation, and colocalized with calnexin and Lamp1 [55]. S-acylation of P1X7R thus involves multiple C-terminal cysteine residues and controls the channel surface expression and association with lipid rafts.

### Store-operated Ca^2+^ channels

#### Orai1

The Ca^2+^-selective ion channel ORAI1 (Table 1), encoded by the *ORAI1* gene, is the pore component of the store-operated Ca^2+^ entry pathway. This Ca^2+^ Release-Activated Channel (CRAC) mediates intracellular signals that control the proliferation of T cells and loss-of-function mutations in *ORAI1* cause severe combined immunodeficiencies. Effective immune responses require Ca^2+^ entry through ORAI1 when T cell receptors interact with antigen-presenting cells at the immune synapse (IS) [56]. We and others have shown by H^3^-palmitate and PEG incorporation that ORAI1 is S-acylated at cysteine residue C143 (Figure 2) and that this PTM positively regulates store-operated Ca^2+^ entry [57,58]. ORAI1 S-acylation by zDHHC20 targets the Ca^2+^ channel to lipid rafts, enhancing the recruitment and signalling of T-cell receptors at the IS in Jurkat T cells. The S-acylation-deficient ORAI1 C143A mutant mediated reduced CRAC currents and was poorly recruited to the IS, severely impairing synapse formation and T cell activation. S-acylation of ORAI1 thus controls the recruitment and function of channels and receptors at the IS to mediate efficient T cell responses.

#### STIM1

The stromal interaction molecule 1 (STIM1, encoded by the *STIM1* gene) is an ER-resident protein with a single transmembrane domain that, upon Ca^2+^ depletion of the ER, undergoes a series of conformational changes that promote its translocation to cortical ER structures, where it traps and gate ORAI Ca^2+^-permeable channels. STIM1 is primarily expressed in T cells and in skeletal muscles [59] and as ORAI1 is critical for T cells activation [60] and muscle development [61]. Using Acyl-RAC, STIM1 was shown to be S-acylated on cysteine 437 (Figure 2) in Jurkat T cells and HEK-293 cells. This S-acylation is required for a fully functional SOCE and for efficient STIM1–ORAI1 *puncta* formation and colocalization [62], indicating that S-acylation positively regulates STIM1 activation step(s).

### Ca^2+^ pumps and exchangers

#### NCX1

The sodium-calcium exchanger 1 (NCX1, encoded by the *SLC8A1* gene) is a prototypical case of regulation of a Ca^2+^ transporter by S-acylation (Table 1). NCX1 catalyses the bidirectional, electrogenic exchange of one Ca^2+^ for three Na^+^ ions, driven by the transmembrane sodium gradient. NCX1 shapes Ca^2+^ signals that control the contractility of cardiac muscle and alterations in the Ca^2+^ extruding activity of NCX1 are associated with cardiac ischaemia, heart failure, and arrythmia. NCX1 is S-acylated on a single residue, C739 (Figure 2), a process regulated by a distal amphipathic helix (aa 740–756) that directly interacts with DHHC–PATs [63]. This reversible PTM (the only reported so far for NCX1) enhances the inactivation of the exchanger [64]. Subsequent reports by the Fuller group identified zDHHC5 and APT-1 as the enzymes mediating dynamic NCX1 S-acylation and de-acylation at the plasma membrane, respectively, and revealed that S-acylation increases the affinity of NCX1 for ordered lipids and for its endogenous exchange inhibitory peptide (XIP) [65]. 2-BP and the C739A mutation decreased intermolecular fluorescence resonance energy transfer (FRET) between tagged NCX1 without altering the monomer/dimer ratio, indicating that S-acylation enhances interactions between NCX1 protomers. Pharmacological manipulations of cellular cholesterol altered FRET signals of WT but not of non-S-acylated NCX1-C739A, which partitioned in cholesterol-poor domains in giant plasma membrane vesicles, indicating that S-acylation controls the affinity of NCX1 for lipid rafts. Biochemically, 2-BP and the C739A mutation decreased the ability of biotinylated XIP to pull-down NCX1 by interacting with a binding site (709–728) within the NCX1 regulatory intracellular loop. Functionally, the C739A mutation enhanced the resting cytosolic Ca^2+^ concentration in HEK-293 cells by augmenting Na^+^-driven Ca^2+^ entry across the exchanger. This comprehensive study establish that S-acylation controls the inactivation of the exchanger by enhancing its affinity for lipids and for its endogenous inhibitory motif. The recently reported cryo-EM structures of human cardiac NCX1 [66] will provide insight into the structural basis of this modulation which has important functional consequences and clinical implications as dysregulation of NCX1 S-acylation has been linked to heart failure and left ventricular hypertrophy [67].

#### SERCA

The sarco-ER Ca^2+^ pump is an intracellular pump that transports Ca^2+^ from the cytoplasm into the lumen of the ER or, in muscle, SR. SERCA catalyses Ca^2+^ transport across the membrane using the energy derived from ATP hydrolysis. Using acyl-RAC and LC–MS/MS, the SERCA 1A isoform (encoded by the *ATP2A1* gene) was shown to be S-acylated on eight cysteines located throughout its cytoplasmic domain, C12, C344, C349, C364, C471, C636, C674 and C675 (Figure 2) [68]. Out of these eight acylated cysteines, six are conserved in SERCA2 and SERCA3. The functional impact of SERCA S-acylation is currently not known. However, it has been observed that 2-BP treatment leads to enrichment of SERCA2b at ER-mitochondrial contact sites, indicating that S-acylation might regulate SERCA localization [69].

### Ca^2+^ release channels

#### IP3R1

The inositol 1,4,5-trisphosphate receptor is an intracellular channel that releases Ca^2+^ from the ER into the cytosol upon binding inositol trisphosphate (InsP3) (Table 1). IP3R converts external cues into intracellular Ca^2+^ signals and plays a fundamental role in a wide spectrum of cellular processes in both excitable and non-excitable cells. Mammals express three distinct IP3R isoforms (type 1–3, encoded by the *ITPR1-3* genes) that have overlapping yet distinct expression patterns with most of the cells expressing more than one isoform [70]. Loss-of-function mutations in IP3R1 lead to neurological disorder while loss-of-function mutation in IP3R2 and IP3R3 lead to exocrine disorders and cancer [71]. Using Acyl-biotin exchange and MS from rat overexpressing ITPR1, Cys 56 and 849 and potentially 2214 of IP3R1 (Figure 2) were identified as S-acylated [72]. Their substitution reduced S-acylation by 75%, suggesting that other cysteines might be S-acylated. Genetic invalidation of the ER-localized selenium-containing protein, selenoprotein K (Selk), or of its associated PAT zDHHC6 decreased IPTR1 S-acylation and impaired receptor stability and InsP3-mediated Ca^2+^ release. S-acylation by an ER-bound zDHHC6/Selk complex thus promotes the stable expression and function of IP3R1.

#### Ryanodine receptor

The ryanodine receptors (RyRs) are another class of Ca^2+^-release channels (Table 1) comprising three isoforms (RyR1-3, encoded by the *RYR1-3* genes) that orchestrate muscle contraction [73] and neurotransmitter release [74]. Malfunctions in RyRs have been linked to the onset of myopathies, diabetes, and neurodegenerative diseases [73]. Among the isoforms, RYR1 is primary expressed in skeletal muscle, RYR2 predominates in myocardium, while RYR3 is more widespread with expression in the brain [75]. Using acyl-RAC, LC–MS/MS, and palmitate incorporation, RyR1 was found to be S-acylated on 18 distinct cysteine residues distributed within multiple functional domains (Figure 2) [68]. Preventing S-acylation with hydroxylamine reduced ryanodine binding in intact SR vesicles while 2-BP reduced stimulus-coupled Ca^2+^ release in mouse myofibers [68]. S-acylation of RyR1 at multiple sites thus enhances the SR Ca^2+^ release and might promote excitation-contraction coupling in skeletal muscle.

## Conclusion

In this review, we summarize recent studies addressing the molecular basis and functional consequence of the S-acylation of Ca^2+^ transport proteins, a rapidly evolving field. From our inventory, it appears that no straight prediction can be made as to the expected effects of S-acylation on the function of a Ca^2+^ transport protein as summarized in Figure 2 and Table 1. While positive modulation of transport activity is frequently reported, several transporters are negatively modulated by S-acylation and opposite effects have been reported to result from the S-acylation of distinct residues on the same transport protein. Gains of function are mostly linked to an enhanced trafficking of the transport proteins or to their increased interactions with membrane lipids, a prominent feature. However, S-acylation has been reported to either target or remove transport proteins from lipid rafts, and the mere accumulation of transporters in these lipid domains does not necessary implies a gain of function.

A better understanding of the dynamic impact of S-acylation on the structural constraints of Ca^2+^ transport by membrane proteins is therefore required to model the consequences of this regulation for cellular Ca^2+^ fluxes. Currently, over 40 ion channel subunits, including some Ca^2+^ channels mentioned in this review, have been experimentally confirmed to undergo S-acylation. Advances in proteomics will likely increase the number of S-acylated Ca^2+^ signalling molecules identified. To predict the functional consequences of S-acylation on Ca^2+^ transport, several points must be addressed: (1) developing tools for time-resolved analysis of protein palmitoylation, to better relate the PTM with alterations in Ca^2+^ transport, (2) designing selective inhibitors of individual zDHHC enzymes, to establish the molecular basis of (potentially) multiple S-acylation cascades, as the widely used inhibitor 2-BP has several off-target effects on enzymes involved in lipid metabolism, (3) mapping the spatiotemporal pattern of S-acylation during a transport protein life cycle, (4) modelling the effect of S-acylation on distinct steps of Ca^2+^ transport, to move beyond the simplistic ‘membrane anchor’ model, (5) exploring the complex interactions between S-acylation and other PTMs such as phosphorylation, and (6) examining the functional consequences of S-acylation at both the cellular and organism levels, with the aim to unveil novel therapeutic strategies. Future research should aim to uncover the structural constrains imposed by S-acylation on the fluxes of ions across these membrane proteins.

## Perspectives

- S-acylation is a dynamic PTM that regulates the activity of Ca^2+^ transport proteins driving neurotransmission, cardiac contraction, and immune responses.
- S-acylation modulates the activity of Ca^2+^ pumps, channels, and exchangers by altering their conformation and interactions with membrane lipids.
- Establishing the structural constrains imposed by S-acylation on the activity of Ca^2+^ transporters could be exploited to treat neuronal, cardiac and immune diseases.

## Open Access

Open access for this article was enabled by the participation of University of Geneva in an all-inclusive *Read & Publish* agreement with Portland Press and the Biochemical Society under a transformative agreement with Individual.

## Abbreviations

CNS: central nervous system
CRAC: Ca^2+^ Release-Activated Channel
ER: endoplasmic reticulum
FRET: fluorescence resonance energy transfer
HD: Huntington's disease
IS: immune synapse
NMDA: *N*-methyl-d-aspartate
17-ODYA: 17-octadecynoid acid
PTM: post-translational modification
RyR: Ryanodine receptor
SR: sarcoplasmic reticulum
